# Sex Differences in the Blood Concentration of Tacrolimus in Systemic Lupus Erythematosus and Rheumatoid Arthritis Patients with CYP3A5*3/*3

**DOI:** 10.1007/s10528-017-9795-8

**Published:** 2017-03-21

**Authors:** Ayano Ito, Yuko Okada, Tadahiro Hashita, Tohru Aomori, Keiju Hiromura, Yoshihisa Nojima, Tomonori Nakamura, Takuya Araki, Koujirou Yamamoto

**Affiliations:** 1 Department of Pharmacy, Gunma University Hospital, 3-39-15 Showa-machi, Maebashi, Gunma 371-8511 Japan; 2 Faculty of Pharmacy, Takasaki University of Health and Welfare, 60 Ohrui-machi, Takasaki, Gunma 370-0033 Japan; 3 Department of Clinical Pharmacology, Gunma University Graduate School of Medicine, 3-39-22 Showa-machi, Maebashi, Gunma 371-8511 Japan; 4 Center for Medical Education, Gunma University Graduate School of Medicine, 3-39-22 Showa-machi, Maebashi, Gunma 371-8511 Japan; 5 Department of Medicine and Clinical Science, Gunma University Graduate School of Medicine, 3-39-22 Showa-machi, Maebashi, Gunma 371-8511 Japan; 6 Department of Pharmacy, NTT Medical Center Tokyo, 5-9-22 Higashi-Gotanda, Shinagawa-ku, Tokyo, 141-8625 Japan; 7 Department of Clinical Pharmacy, Graduate School of Pharmaceutical Sciences, Nagoya City University, 3-1 Tanabe-dori, Mizuho-ku, Nagoya, 467-8603 Japan; 8 Center for Social Pharmacy and Pharmaceutical Care Sciences, Division of Pharmaceutical Care Sciences, Keio University Faculty of Pharmacy, 1-5-30 Shibakoen, Minato-ku, Tokyo, 105-8512 Japan

**Keywords:** Tacrolimus, Sex difference, CYP3A5, CYP3A4, ABCB1

## Abstract

The purpose of this study was to describe the impact of sex and cytochrome P450 3A5 (CYP3A5) variant on the blood concentration of tacrolimus in patients with systemic lupus erythematosus or rheumatoid arthritis. The blood concentration of tacrolimus (ng/mL) divided by the daily dose of tacrolimus (mg/day) and the patient’s weight (kg) (C/D) was obtained from 55 patients. The C/D value was analysed according to genetic variation in *CYP3A5* or ATP binding cassette subfamily B member 1 (*ABCB1*), sex, and age. The C/D value in the CYP3A5*3/*3 group was significantly higher than in the CYP3A5*1/*1 and *1/*3 groups (*p* < 0.05, effect size: *d* = 1.40). In the CYP3A5*3/*3 group, the concentration of tacrolimus was significantly higher in men than in women (*p* < 0.05, effect size: *d* = 1.78). Furthermore, in the CYP3A5*3/*3 group, the concentration of tacrolimus was significantly higher in women aged over 50 years than in women aged under 50 years (*p* < 0.05, effect size: *d* = 1.18). In contrast, *ABCB1* genetic variations did not show any significant effect on the C/D value. Since the blood concentration of tacrolimus in patients with CYP3A5*3/*3 varies depending on sex and age, these factors should be considered when studying the difference of sex in CYP3A.

## Introduction

Tacrolimus, an immunosuppressant, is used in transplantation and for the treatment of autoimmune diseases, such as rheumatoid arthritis (RA) and systemic lupus erythematosus (SLE) (Anderson 2005; Bao et al. 2008; Kawai et al. 2011; Miyasaka et al. 2009). The pharmacokinetic (PK) profile of tacrolimus is known to vary greatly between individuals. Thus, therapeutic drug monitoring is recommended for maintaining the concentration of tacrolimus within the therapeutic range to obtain sufficient efficacy and avoid severe adverse effects.

Orally administered tacrolimus is mainly metabolised by cytochrome P450 (CYP) 3A4 and 3A5 in the liver and intestine (Dai et al. 2006; Iwasaki 2007) and is transported out of cells via ATP binding cassette subfamily B member 1 (ABCB1) (Saeki et al. 1993). Recently, the impact of differences in the activity of CYP3A5 on the PK profile of tacrolimus has been focussed upon because CYP3A5 accounts for more than 50% of total CYP3A activity in wild-type CYP3A5 carriers and the activity of CYP3A5 is affected strongly by variants. Specifically, the 6986A > G variant in intron 3 of *CYP3A5* (CYP3A5*3) (rs776746), is known as one of the most important single nucleotide polymorphisms (SNPs) in *CYP3A5*, and patients harbouring homozygous CYP3A5*3 have a complete deficiency of CYP3A5 expression due to improper splicing of its mRNA (Hustert et al. 2001; Kuehl et al. 2001).

Recently, the influence of CYP deficiency due to variants has been considered to be one of the causes of unexpected drug interactions, especially for medicines metabolised by several kinds of CYPs. For instance, in patients with impaired CYP2C19 activity, the plasma concentration of voriconazole, which is a substrate of CYP2C9, 2C19, and 3A4, was strongly affected by the co-administration of CYP3A4 inhibitors compared to patients with normal CYP2C19 activity (Shi et al. 2010). Similarly, in patients with impaired CYP3A5 activity, the PK profile of tacrolimus is expected to be strongly affected by inter-individual differences in factors modulating CYP3A4 activity, such as age, the concomitant administration of a CYP3A4 inhibitor or enhancer, and variants. Some studies have reported that CYP3A4 activity was significantly higher in the liver of women than in men (Diczfalusy et al. 2011; Wolbold et al. 2003). Chen et al. also reported that the area under the curve (AUC) of midazolam, a substrate of CYP3A4, was lower in women than in men (Chen et al. 2006). These data suggest that sex might be a modulating factor of CYP3A4 activity and affects the PK profile of tacrolimus in patients with CYP3A5 deficiency.

In this study, we assessed the impact of sex on the PK profile of tacrolimus in SLE and RA patients with CYP3A5*3/*3.

## Materials and Methods

### Patients

We enrolled 55 unrelated Japanese patients (11 males and 44 females) treated with a once-daily low dose of oral tacrolimus for SLE or RA at Gunma University Hospital between 2007 and 2011 in this study. Patients using concomitant drugs which are strong inhibitors of CYP3A4 activity, such as azole antifungal drugs, and treating with hormone therapy were excluded from this study. Patients who were screened less than three times for tacrolimus concentration were also excluded. Written consent was obtained from all patients after they had been informed of the experimental procedure and the purpose of this study. Approval for this study was obtained from the Institutional Review Board of Gunma University Hospital and the Ethical Committee for Human Genome Analysis at Gunma University.

### Genotyping

Genomic DNA was isolated from peripheral blood using a QIAamp DNA Mini Kit (QIAGEN, Hilden, Germany). The CYP3A5*3 and *ABCB1*:c.3435T > C (rs1045642) (Hodges et al. 2011) variants were detected by the polymerase chain reaction-restriction fragment length polymorphism (PCR–RFLP) method according to previous reports with slight modifications (Miao et al. 2008; Tang et al. 2002). Briefly, the PCR products were digested with *Ssp*I for CYP3A5*1 variant reported as functional CYP3A5 (Lamba et al. 2012) and CYP3A5*3 or *Dpn*II for *ABCB1*:c.3435T > C. The *ABCB1*:c.2677G > T/A (rs2032582) (Hodges et al. 2011) mutation was determined by the standard Sanger sequencing method. The details of the oligonucleotide primers used and PCR product sizes are presented in Table 1.

**Table 1:** Conditions for genotype analysis of *CYP3A5* and *ABCB1*

SNPs	Primer sequences										PCR product	Reference
CYP3A5*3	Forward:	5′-	CAT	CAG	TTA	GTA	GAC	AGA	TGA	-3′	293 bp	Miao et al. 2008
	Reverse:	5′-	GGT	CCA	AAC	AGG	GAA	GAA	ATA	-3′		
*ABCB1*:c.3435T > C	Forward:	5′-	GAT	CTG	TGA	ACT	CTT	GTT	TTC	-3′	244 bp	Miao et al. 2008
	Reverse:	5′-	GAA	GAG	AGA	CTT	ACA	TTA	GGC	-3′		
*ABCB1*:c.2677G > T/A	Forward:	5′-	GCA	GGC	TAT	AGG	TTC	CAG	GCT	-3′	224 bp	Tang et al. 2002
	Reverse:	5′-	TGA	GGA	ATG	GTT	ATA	AAC	ACA	-3′		

### Determination of the Blood Concentration of Tacrolimus

Whole-blood samples were obtained from patients at 12 h after the administration of tacrolimus and were treated with EDTA-2K to prevent coagulation. Concentrations of tacrolimus are measured routinely using Dimension EXL with LM (SIEMENS, Munich, Germany). The assay for tacrolimus utilises the affinity column-mediated immunoassay (ACMIA) and mixing or lysis of whole-blood samples are automatically treated by Dimension system. Before measurement every morning, the coefficients of variation are adjusted within 10% using precision control products of three concentrations (L: 4.5, M: 11, H: 22 ng/mL). The interday variations using Dimension EXL with LM were as follows: Control L, 9.5%; Control M, 9.7%; Control H, 10.0%. The blood concentration of tacrolimus (ng/mL) divided by the daily dose of tacrolimus (mg/day) and the patient’s weight (kg) is shown as a C/D value and used as the PK index.

### Statistical Analysis

Deviation from the Hardy–Weinberg equilibrium was assessed by the Chi-square test. Differences between sexes in parameters of the patients were compared using Student’s *t* test or *χ* ^2^ test. The effect of genetic variation on the C/D value was assessed by Student’s *t* test, and the effect of sex on the C/D value was assessed by two-way factorial analysis of variance with Tukey’s HSD post hoc test for multiple comparisons using SPSS Statistics version 20.0 (IBM Japan, Tokyo, Japan). Post hoc power (1 − β) was calculated by G*power3. A *p* value less than 0.05 was considered to indicate a statistically significant difference in all analyses. All *p* values were assessed with two-tailed tests, and 95% confidence intervals (CIs) were calculated between the groups.

## Results

The characteristics of the patients, including the allele frequencies of CYP3A5*3, *ABCB1*:c.3435T > C, and *ABCB1*:c.2677G > T/A are summarised in Table 2. The distributions of all genetic variations were in Hardy–Weinberg equilibrium. No significant differences in age, hepatic or renal function, or other clinical data were found among each variant.

**Table 2:** Characteristics of the patients

Parameters	Men	Women	*p* value
Number of subjects *n* (%)	11 (20)	44 (80)	–
Age			
Range (median), years	25–80 (61)	19–81 (43.5)	0.12^a^
Body weight			
Range (median), kg	44–75 (64.2)	40–71 (50)	<0.05^a^
Disease *n* (%)			
RA	7 (63.6)	14 (31.8)	0.03^b^
SLE	4 (36.4)	30 (68.2)	
*CYP3A5 n* (%)			
*1/*1	1 (9.1)	3 (6.8)	0.50^b^
*1/*3	6 (54.5)	20 (45.5)	
*3/*3	4 (36.4)	21 (47.7)	
*ABCB1*:c.3435T > C *n* (%)			
CC	7 (63.6)	19 (43.2)	0.09^b^
CT	4 (36.4)	13 (29.5)	
TT	0	12 (27.3)	
*ABCB1*:c.2677G > T/A *n* (%)			
GG	4 (36.4)	6 (13.6)	0.08^b^
GT	3 (27.3)	10 (22.7)	
GA	3 (27.3)	7 (15.9)	
TA	1 (9.1)	8 (18.2)	
TT	0	11 (25.0)	
AA	0	2 (4.5)	

^a^Student’s *t* test

^b^ *χ* ^2^ test

**Fig. 1:**
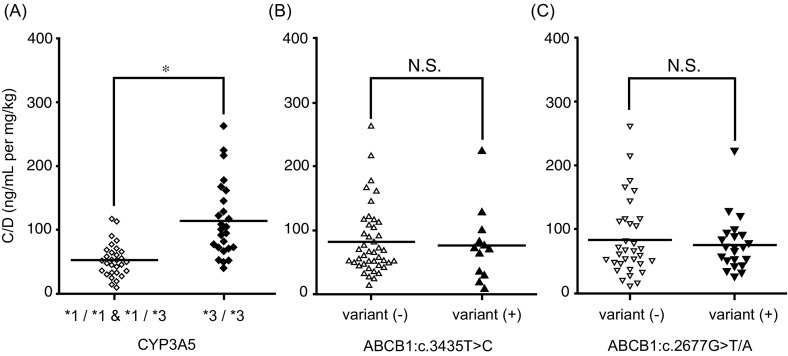
Effect of *CYP3A5* (**a**), *ABCB1*:c.3435T > C (**b**), and *ABCB1:c.*2677G > T/A (**c**) variants on the C/D value of tacrolimus. The *open symbols* indicate the wild-type and heterozygous alleles, and the *closed symbols* indicate the mutant alleles. *Each bar* indicates a median value. *p < 0.05. *NS* not significant

The C/D value was significantly higher in the CYP3A5*3/*3 patients than in the others (*CYP3A5* *1/*1 and *1/*3: mean 52.1, 95% CI 42.8–61.4; CYP3A5*3/*3: mean 114.8, 95% CI 91.7–137.9) (*p* < 0.05, effect size: *d* = 1.40, Fig. 1a). Conversely, *ABCB1* genetic variations had an insignificant effect on the C/D value (*ABCB1*:c.3435T > C variant (−): mean 82.2, 95% CI 66.3–98.0; variant (+): mean 76.9, 95% CI 44.0–109.6; Fig. 1b) (*ABCB1*:c.2677G > T/A variant (−): mean 84.1, 95% CI 63.6–104.5; variant (+): mean 76.4, 95% CI 58.2–94.6; Fig. 1c).

**Fig. 2:**
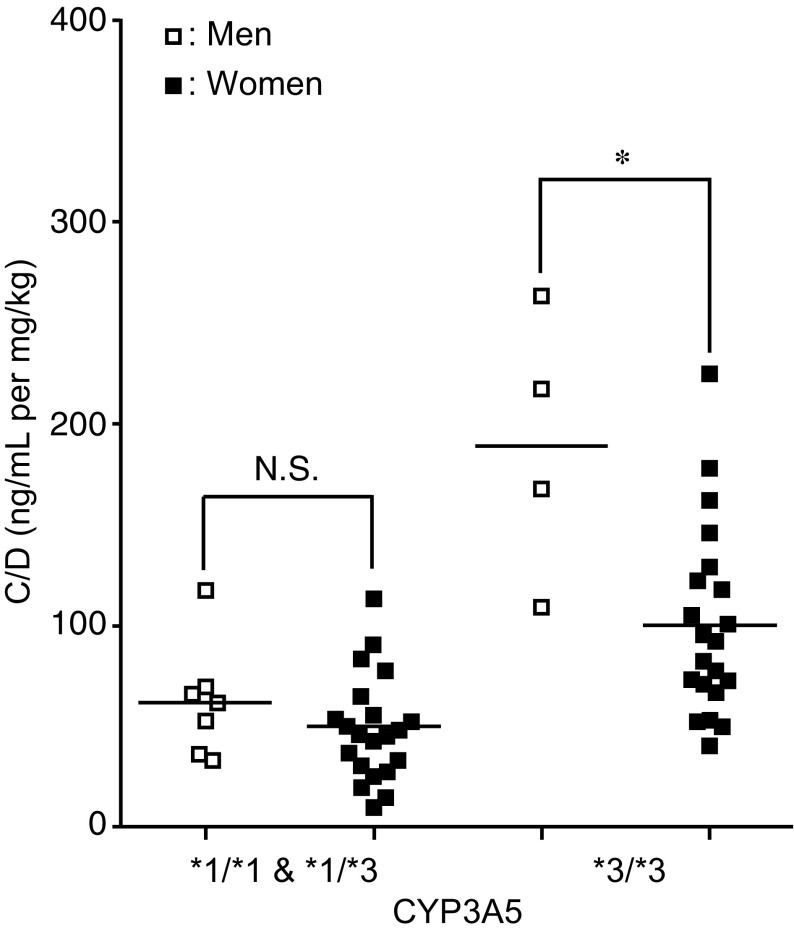
Effect of sex on the C/D value of tacrolimus with respect to *CYP3A5* variants. The *open and closed squares* indicate men and women, respectively. *Each bar* indicates a median value. *p < 0.05. *NS* not significant

Although there were no sex differences in the C/D value when all of the samples were considered, the C/D value was significantly lower in women than in men in the patients with CYP3A5*3/*3 (women: mean 100.6, 95% CI 81.0–120.2; men: mean 189.2, 95% CI 133.0–245.5) (*p* < 0.05, effect size: *d* = 1.78, Fig. 2). Sex differences in the C/D value of tacrolimus were not found for any of the *ABCB1* variants (data not shown).

**Fig. 3:**
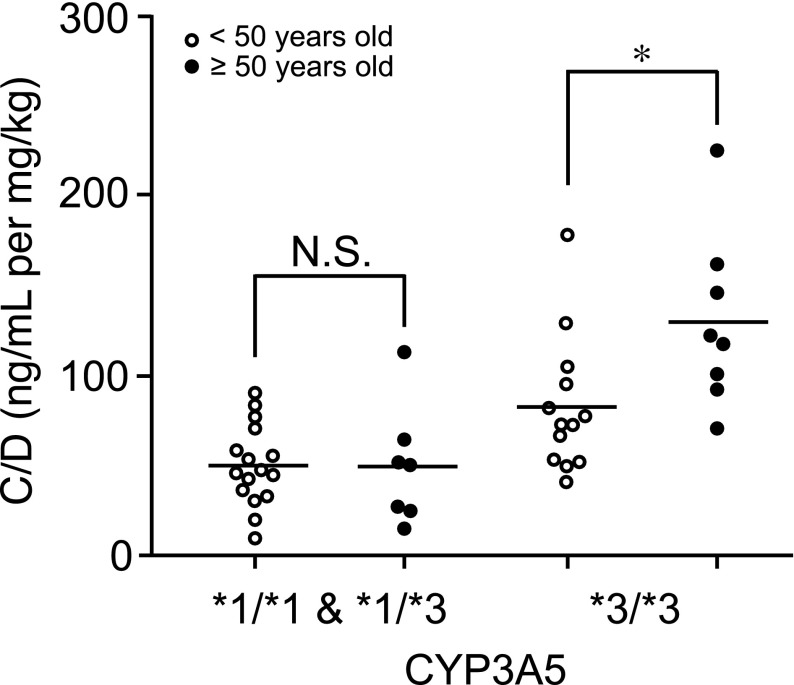
Effect of age on the C/D value of tacrolimus in women with respect to *CYP3A5* variants. The *open and closed circles* indicate patients aged under 50 years and over 50 years, respectively. *Each bar* indicates a median value. *p < 0.05. *NS* not significant

In women in the CYP3A5*3/*3 variant group, the C/D value was significantly higher in those aged over 50 years than in those aged under 50 years (over 50 years of age: mean 129.6, 95% CI 98.4–160.8; under 50 years of age: mean 82.8, 95% CI 63.1–102.4) (*p* < 0.05, effect size: *d* = 1.18, Fig. 3). However, differences between the CYP3A5*1/*1 and *1/*3 variant groups were not found (>age 50 years: mean 44.7, 95% CI 21.3–68.1; <age 50 years: mean 52.8, 95% CI 42.4–63.0; Fig. 3).

## Discussion

Orally administered tacrolimus is known to be metabolised by CYP3A5 in the liver and intestine (Dai et al. 2006; Iwasaki 2007) and excreted by P-gp (Saeki et al. 1993). It is also known that the blood concentration of tacrolimus is increased by its interaction with drugs which inhibit CYP3A4 activity (Dai et al. 2006; Iwasaki 2007), indicating that tacrolimus is metabolised by CYP3A4 at least partly. In this study, we targeted SLE and RA patients taking low-dose tacrolimus, and we found that the C/D value was significantly different according to the sex of patients with CYP3A5*3/*3. To the best of our knowledge, this is first study to demonstrate sex differences in the blood concentration of tacrolimus in patients with CYP3A5*3/*3.

Velicković-Radovanović et al. also reported that the AUC of tacrolimus after oral administration was significantly larger in men than in women (Velickovic-Radovanovic et al. 2012). Using 450 transplant recipients, Stratta et al. showed that the metabolism of tacrolimus was slower in men than in women and suggested that the dose of tacrolimus should be adjusted based on sex (Stratta et al. 2011).

Conversely, in most population PK studies, sex is excluded as a candidate factor which can affect the PK profile of tacrolimus (Diczfalusy et al. 2011; Miao et al. 2008; Tang et al. 2002). As the reason for these discrepancies in the existence of sex differences in the PK profile or clinical efficacy of tacrolimus, Ohtani et al. suggested that more than 600 cases are required to detect sex differences in the activity of CYP3A4, although 50 cases were sufficient to detect the effect of CYP3A5 variants (Ohtani et al. 2011). Therefore, we hypothesised that a small sample can be used to evaluate the influence of CYP3A4 only when a *CYP3A5*-deficient group is analysed.

In this study, sex differences were recognised in only the CYP3A5*3/*3 group, although no differences were observed when all groups were analysed. We considered that this was because tacrolimus is metabolised by CYP3A4, the activity of which differs by sex, but not CYP3A5 in the CYP3A5*3/*3 group. Sex differences in the hepatic expression of CYP3A4 have been reported in some in vitro and in vivo studies. In 2003, Wolbold et al. reported that CYP3A4 mRNA levels were twofold higher in the liver of women than that of men (Wolbold et al. 2003). Diczfalusy et al. also reported sex differences in the activity of human CYP3A by using 4beta-hydroxycholesterol (Diczfalusy et al. 2011). In addition, several reports described the possibility of sex differences in the PK profiles of CYP3A4 or CYP3A5 substrates (Chen et al. 2006; Harris et al. 1995). These reports support our data and opinion. Indeed, recent data have suggested that the female-predominant expression of CYP3A4 is due to the inherent, sex-dependent suboptimal activation of transcription networks responsible for the hormone-induced expression of the isoform in men (Choi et al. 2013; Thangavel et al. 2013). This report also supports our data and opinion.

Furthermore, we found an age-related difference in the PK profile of tacrolimus in female patients harbouring CYP3A5*3/*3. Generally, the average age of menopause is approximately 50 years, and the levels of female hormones decrease after menopause. Thus, we hypothesised that the age-associated change of female hormone levels affected CYP3A4 expression and caused the age-related difference in the PK profile of tacrolimus in female patients with impaired CYP3A5 activity.

Although ACMIA method has cross-activity with tacrolimus metabolites in this study, we did not analyse the concentration of metabolites mediated by CYP3A4 or CYP3A5. Thus, we could not conclude that sex differences in CYP3A4 activity were the cause of the sex differences in the PK profile of tacrolimus in patients with CYP3A5*3/*3. Although a hypothesis also exists in which the sex differences in the PK profile of tacrolimus are the result of clearance, we suggest that analysis of metabolite levels would resolve this issue. Furthermore, it is difficult to register new male patients except eleven patients in this study although the number of male patients was very small. Because the sex ratio in SLE and RA are 1:9 and 1:4, almost none of male patients exist in these female-specific disorders. We recognise this to be a pilot study, and that larger numbers of patients are needed to allow more detailed analysis.

A number of previous studies of sex difference are reported that the clinical importance is rare, although the clearance of CYP3A-mediated drug is higher in women than in men(Cotreau et al. 2005; Greenblatt and von Moltke 2008). However, no study of sex difference featured CYP3A5 genetic variation has performed. We suggest that the cause regarded as infrequently clinical effects was performed without categorisation of CYP3A5 genetic variation and thus sex differences in previous studies were underestimated.

In conclusion, we targeted SLE and RA patients treated with low-dose tacrolimus and we identified sex differences in the C/D values, especially in women; the C/D values were significantly related to age in patients with CYP3A5*3/*3. Although significant differences were observed in this study, more patients are needed to verify the appropriateness of this observation due to insufficient male sample size.
